# 

**Published:** 1842-06-25

**Authors:** 


**CONTENTS OF No. 26.**

Dr. William P. Johnston's Case of Bright's Disease—with Remarks, - 401

ANALECTA: Dr. Marshall Hall's Observations on the Prevention and Treatment of Apoplexy and Hemiplegia, - - - - 405

Plethora, or Fulness, - - - - - - - 406

Inanition, - - - - - - - - - - 407

Dyspepsia and Cachexia, - - - - - - 408

Gout, - - - - - - - - - - 408

Disease of the Heart, - - - - - - - 409

Disease of the Capillary and Minute Vessels, - - - 409

Muscular Efforts, - - - - - - - - 409

Dr. Robert Storr's Case of Venous Hæmorrhage from an Abscess resulting from Erysipelas Phlegmonoides, - - 410

Alfred M'Clintock, Esq., on the Employment of the Chloride of Zinc as an Escharotic, - - - - - - - 411

M. Andral on the Changes of the Blood in Disease, - - 413

Sanguineous Temperament, - - - - - - - 413

The Lymphatic Temperament and Anæmia, - - - 413

Mr. A. N. Ruddock's Case of Penetrating Wounds of both Lungs—Recovery, - - - - - - - - - 414

Hæmorrhage from Polypus Uteri, - - - - - - 416

Use of Drastic Purgatives, - - - - - - - 416

